# Silk Fibroin‐Stabilized Lapachol Microemulsion Enhances Antiglioma Activity In Vitro

**DOI:** 10.1002/cbdv.202501414

**Published:** 2026-01-08

**Authors:** Jardel P. Queiroz, Fábio R. Oliveira, Eline Gomes Santos, Fabrício Holanda, Victor Marinho, Edilene Oliveira da Silva, José Carlos T. Carvalho, Caio P. Fernandes, Barbarella M. Macchi, Irlon M. Ferreira, José Luiz M. Nascimento

**Affiliations:** ^1^ Programa De Pós‐Graduação Em Farmacologia e Bioquímica Universidade Federal do Pará Belém Brazil; ^2^ Programa De Neurociências e Biologia Celular Universidade Federal do Pará Belém Brazil; ^3^ Laboratório De Neuroquímica Molecular e Celular Instituto De Ciências Biológicas Universidade Federal do Pará Belém Brazil; ^4^ Laboratório De Biocatálise e Síntese Orgânica Aplicada Curso De Química Universidade Federal do Amapá Macapá Brazil; ^5^ Departamento De Ciências Biológicas e Da Saúde Laboratório De Pesquisa em Fármacos Curso De Farmácia Universidade Federal do Amapá Macapá Brazil; ^6^ Laboratório De Biologia Estrutural Instituto De Ciências Biológicas Universidade Federal do Pará Belém Brazil; ^7^ Instituto Nacional de Ciência e Tecnologia Em Neuroimunomodulação (INCT‐NIM) Rio de Janeiro Brazil; ^8^ Instituto Nacional de Ciência e Tecnologia De Biologia Estrutural e Bioimagem (INCT‐INBEB) Rio de Janeiro Brazil; ^9^ Departamento De Ciências Biológicas e Da Saúde Laboratório De Controle de Qualidade e Bromatologia Curso De Farmácia Universidade Federal do Amapá Macapá Brazil

**Keywords:** anticancer therapy, controlled release, gliomas, lapachol, nanopharmaceutical, silk fibroin

## Abstract

Gliomas are the most prevalent of the brain tumors, and are associated with high mortality and limited therapeutic options. This study introduces, for the first time, a silk fibroin (SF)‐based microemulsion as a nanocarrier for lapachol (LP). The nanocarrier demonstrated improved stability, selectivity, and antiproliferative efficacy against glioma cells, compared to conventional pharmacological approaches. The aim of this study was to evaluate the therapeutic potential of LP and two nanostructured formulations, a lapachol nanoemulsion (LPN) and an SF‐based microemulsion (LP‐SF), in human (AHOL1) and rat (C6) glioma cells. Both formulations exhibited colloidal stability, with LP‐SF showing sustained drug release and higher cytotoxicity (half‐maximal inhibitory concentration [IC_50_] of 19.96 µg/mL for C6 and 1.7 µg/mL for AHOL1), compared to isolated LP (IC_50_ of 44.7 µg/mL for C6 and 3.15 µg/mL for AHOL1) and LPN (33.9 µg/mL for C6 and 2.3 µg/mL for AHOL1). LP‐SF retained selectivity toward tumor cells, while preserving the viability of healthy cells, confirming its lack of harmful effects. These results highlight LP‐SF as a promising nanoplatform for glioma therapy, combining enhanced antitumor efficacy with safety.

## Graphical abstract

Silk fibroin‐stablized lapachol microformulation antiglioma activity.

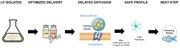

## 1 Introduction

Gliomas, the most common intracranial tumors, account for approximately 81% of malignant brain tumor cases, predominantly affecting men between 50 and 60 years of age. Several hereditary diseases have been linked to an increased risk of glioma (<5% of all glioma cases), including neurofibromatosis types 1 and 2, tuberous sclerosis, Lynch syndrome, Li‐Fraumeni syndrome, and Maffucci syndrome. Additionally, having a family history of glioma in a first‐degree relative diagnosed at a young age is associated with another 5%–10% of cases [1, 2, 3]. Despite significant therapeutic advancements, the treatment of these tumors remains a major challenge due to their high resistance to chemotherapy and the systemic toxicity of available drugs. Natural products display a remarkable diversity in their structure and chemistry, making significant contributions to discoveries in biology and medicine, particularly regarding their therapeutic potential in cancer treatment [4, 5, 6, 7, 8]. The compound, 2‐hydroxy‐3‐(3‐methyl‐2‐butenyl)‐1,4‐naphthoquinone, commonly known as lapachol (LP) [9, 10], is a natural compound that can be extracted from the *Tabebuia impetiginous* tree, which is native to the Amazon rainforest and grows in various other Latin American countries [11, 12]. LP and derivatives have been investigated as chemotherapeutic leads in breast, prostate, melanoma, leukemia, ovarian, colon, renal cancers, and glioblastomas, and also display antileishmanial, anti‐inflammatory, antimicrobial, and antiplasmodial activities [13, 14, 15, 16, 17, 18, 19]. Although LP demonstrates antiproliferative effects against several tumors, including gliomas, translation is limited by its low aqueous solubility and safety concerns at therapeutically relevant doses [20, 21, 22, 23]. Recent evidence demonstrates that this compound has antiproliferative/anti‐migration effects and modulates ncRNA in urothelial models [24], with performance gains upon encapsulation in nanocarriers (e.g., chitosan) [25] and in nanoemulsions/targeted derivatives [26, 27, 28, 29, 30]. Due to the compound's poor solubility/stability and the need for targeted delivery, encapsulation has been explored in recent studies with reports of greater cellular uptake, controlled release (Korsmeyer–Peppas framework), and enhanced selectivity when using nanocarriers [31, 32].

Silk fibroin (SF), a protein from *Bombyx mori*, has emerged as a versatile drug‐delivery matrix, offering intrinsic steric stabilization and tunable release [33, 34, 35, 36]. SF nanoparticles (SFNs) offer a balanced combination of biocompatibility and drug‐loading capacity that makes them a highly competitive nanocarrier for glioma therapy. In terms of cytotoxicity, drug‐loaded and ligand‐targeted SFNs generally achieve superior glioma cell killing compared to poly (lactic‐co‐glycolic acid) (PLGA) and liposomal carriers. This is because their efficient cellular internalization and controlled intracellular release result in lower half‐maximal inhibitory concentration (IC_50_) values at equivalent drug doses [37]. Solid lipid nanoparticles (SLNs) and micelles often suffer from low long‐term stability and premature drug leakage. Mesoporous nanoparticles and hybrid systems may require complex synthesis routes and involve materials with unclear biodegradability or long‐term toxicity. In contrast, these SF‐based microemulsions combine biocompatibility, mechanical robustness, and diffusional control, arising from *β*‐sheet domains. Studies have demonstrated the use of SF carriers for small molecules and photosensitizers in oncology, underscoring their relevance as an experimental platform [38, 39, 40, 41, 42]. Here, for the first time, we report an SF‐based microemulsion as a nanocarrier for LP for use against glioma. We hypothesized that SF‐stabilized microemulsion (LP‐SF) would enhance LP solubility and cellular delivery whilst maintaining a favorable safety profile in vitro.

## 2 Results and Discussion

### 2.1 Physicochemical Characterization of LP

**FIGURE 1:**
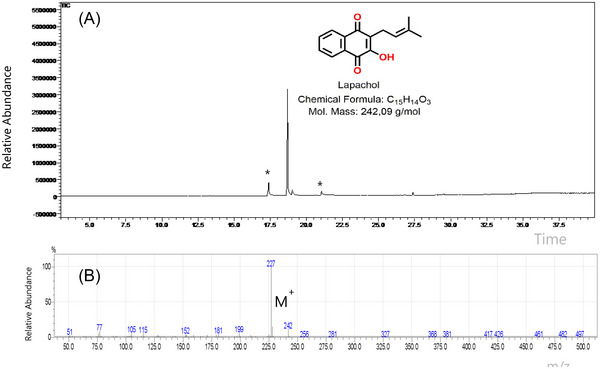
Gas chromatography—mass spectrometry (GC‐MS) analysis of the isolated lapachol: (A) Chromatogram; (B) Mass spectrum.

LP was isolated as a yellowish solid with a melting point of 136–138°C, close to the reference value of 139–140°C [41], confirming adequacy of the purification protocol. Gas chromatography (GC) (Figure 1A) displayed a dominant peak at 18.52 min accounting for 94% of the area, with only trace contaminants (notably free of fatty acids such as oleic and stearic acids). Electron‐impact (EI) MS (70 eV) corroborated the molecular weight (*m/z* 242 [M +]) and yielded the expected base fragment at *m/z* 227 (Figure 1B) [42, 43]. Proton nuclear magnetic resonance (^1^H‐NMR) resonances (Table 1) matched literature assignment [44, 45], including methylene double doublets at *δ* 3.30, vinylic multiplets at δ 5.18–5.23, and aromatic signals at *δ* 7.71 and 8.09, establishing the structure as 2‐hydroxy‐3‐(3‐methyl‐but‐2‐en‐1‐yl)naphthalene‐1,4‐dione (LP). Together, these data verify the compound's identity and high purity, an essential prerequisite for the subsequent formulation and biological assays. Various nanocarriers have been investigated for glioma treatment, including liposomes and polymeric nanoparticles. Importantly, SF offers a combination of biocompatibility, mechanical robustness, and diffusional regulation imparted by its *β*‐sheet domains, which limit water and solute mobility. In this framework, LP‐SF provides electrostatic and protein‐mediated steric stabilization, resulting in a slower and more controlled release profile, together with enhanced antiproliferative activity, compared with free LP and the LP nanoemulsion (LPN) formulation [46, 47, 48].

**TABLE 1:** Analysis of ^1^H nuclear magnetic resonance (NMR) data (in ppm) for lapachol attribution, compared with reports in the literature [44].

^1^H NMR (400 MHz, CDCl_3_)	^1^H NMR (300 MHz, CDCl_3_)
This study	Literature
1.68 (s, 3H)	1.68 (s, 3H)
1.79 (s, 3H)	1.79 (s, 3H)
3.30 (d, *J* = 7.4 Hz, 2H)	3.29 (t, *J* = 0.9, 1H)
—	3.31 (t, *J* = 0.9, 1H)
5.18‐5.23 (m, 1H)	5.17–5.24 (m, 1H)
—	7.34 (s, 1H)
7.71 (dtd, *J* = 30.0, 7.5, and 1.4 Hz, 2H)	7.70 (dddd, *J* =1.2, 7.4, 9.0, and 14.9, 2H)
8.09 (ddd, *J* = 20.4, 7.6, and 1.4 Hz, 2H)	8.08 (dddd, *J* = 1.2, 7.4, 9.0, and 14.9, 2H)

### 2.2 Formulation, Release Kinetics, and Delivery Efficiency

**FIGURE 2:**
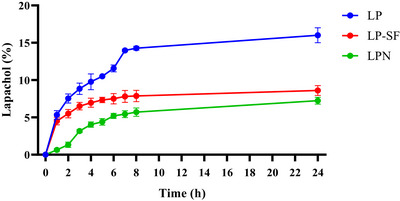
Dialysis release of lapachol (lapachol [LP], lapachol nanoemulsion [LPN], and silk fibroin‐based microemulsion [LP‐SF]). Means ± SD (*n* = 3 independent batches). Statistics: one‐way analysis of variance (ANOVA) with Tukey's post‐hoc test, α = 0.05; symbols indicate comparisons versus LP unless otherwise noted.

Dynamic light scattering (DLS) showed mean diameters of 88.3 ± 1.17 nm for the LPN and 469.86 ± 3.98 nm for the LP‐SF. LP‐SF exhibited a high polydispersity index (PDI) (0.92 ± 0.09), indicating size heterogeneity. The high PDI observed suggests a broad particle size distribution and heterogeneity within the formulation. Such heterogeneity can affect in vivo performance, since nanoparticles of different sizes often follow distinct biological pathways, impacting biodistribution, cellular uptake, and clearance. Nevertheless, our results demonstrate that the SF nanoformulation was effectively internalized by glioma cells and displayed enhanced targeting efficiency. Significantly negative zeta potentials (LPN: −31.5 ± 1.0 mV; LP‐SF: ‐39.8 ± 0.3 mV) are consistent with electrostatic stabilization; in addition, SF affords steric stabilization via protein adsorption and *β*‐sheet networking, likely contributing to colloidal persistence. Over 24 h, LP release was sustained: 8.6% (LP‐SF), 7.2% (LPN), and 16% (LP solution, control), reflecting the same dialysis‐membrane diffusional barrier (Figure 2). Within the first hour, LP‐SF displayed an initial burst of 4.5%, consistent with the release of surface/weakly adsorbed drug, whereas LPN reached 1.3% only by 2 h. Thereafter, the slope decreased toward a quasi‐stationary regime, indicating slower release. The profile was captured by a Korsmeyer–Peppas model with a lag term, with fit (R^2^ = 0.9961); a framework that is frequently applied to polymeric/interfacial carriers in which drug diffusion and matrix/interface relaxation/organization coexist [46, 47]. In SF, increasing *β*‐sheet (Silk II) domains increases crystallinity and hydrophobicity, restricting water/solute mobility and retarding diffusion after the burst, consistent with LP‐SF. Collectively, these data suggest that electrostatic stabilization (negative zeta), combined with steric stabilization (*β*‐sheet network), controls the slower‐release regime (Figure 7). By contrast, in human serum albumin nanoparticles, release is often dominated by desorption from hydrophobic sites and diffusion within the protein network; when labile linkages (e.g., redox‐responsive) are present, relaxation/cleavage of the matrix can also contribute, where these mechanisms are typically less diffusion‐restrictive than the SF β‐sheet–mediated barrier at physiological pH [48].

### 2.3 Antiproliferative Activity

**FIGURE 3:**
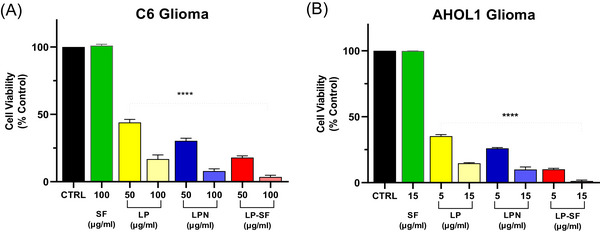
(A) Cell viability test (3‐(4,5‐dimethylthiazol‐2‐yl)‐2,5‐diphenyltetrazolium bromide [MTT]) after 72 h in C6 cells treated with lapachol (LP), lapachol nanoemulsion (LPN), silk fibroin‐based microemulsion (LP‐SF), and SF. LP‐SF showed the lowest cell viability (IC_50_ of 19.96 µg/mL), indicating a higher cytotoxicity than LP and LPN (p < 0.001). (B) Cell viability test (MTT) in AHOL 1 glioma cells treated for 72 h with LP, LPN, LP‐SF, and SF. Data are presented as means ± SD (*n* = 3 independent experiments; three technical replicates each). ****p < 0.0001, compared to the untreated control group (CTRL).

**FIGURE 4:**
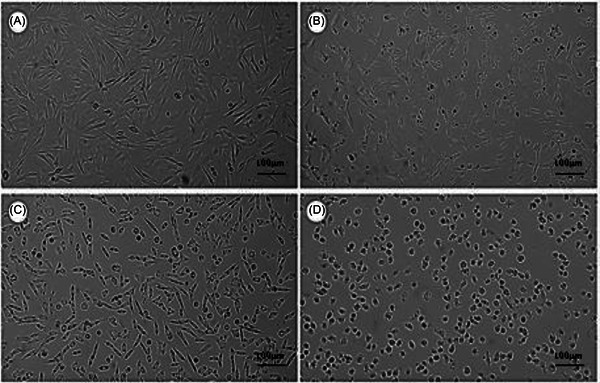
(A) C6 cell proliferation. (B) Cellular morphology after treatment with 100 µg./mL^−1^ lapachol (LP). (C) Treatment with 100 µg.mL^−1^ of lapachol nanoemulsion (LPN). (D) Treatment with 100 µg.mL^−1^ of silk fibroin‐based microemulsion (LP‐SF). The treatments were performed for 72 h. Cells were examined using a Leica DMI6000B phase‐contrast microscope, with images acquired using a 10× objective at 100× magnification with a scale bar of 100 µm.

**FIGURE 5:**
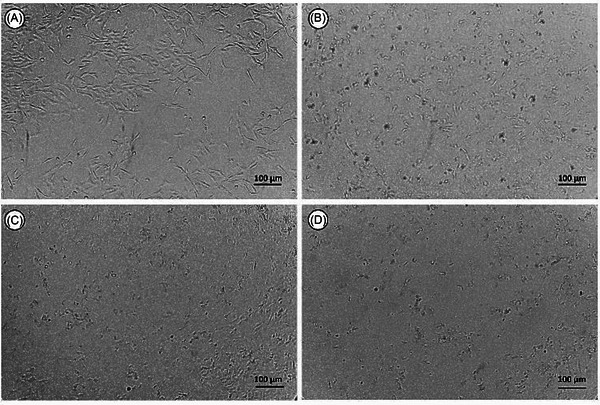
(A) AHOL1 cell morphology. (B) Cell morphology after treatment with 5 µg.mL^−1^ lapachol (LP). (C) Treatment with 5 µg.mL‐1 lapachol nanoemulsion (LPN). (D) Treatment with 5 µg.mL^−1^ silk fibroin‐based microemulsion (LP‐SF). The treatments were performed for 72 h. Cells were examined using a Leica DMI6000B phase‐contrast microscope, with images acquired using a 10× objective at 100× magnification with a scale bar of 100 µm.

3‐(4,5‐dimethylthiazol‐2‐yl)‐2,5‐diphenyltetrazolium bromide (MTT) assays (24–72 h; 10–100 µg mL^−1^) were performed in C6 (rat glioma) and AHOL1 (human glioma) cells. Data are means ± SD from *n* = 3 independent experiments (3 technical replicates each). IC_50_ values were obtained by non‐linear regression (95% CI in SI); group comparisons used one‐way analysis of variance (ANOVA) + Tukey (α = 0.05). Both formulations potentiated LP's antiproliferative effect. IC_50_ values decreased from 44.7 µg mL^−1^ (C6) and 3.15 µg mL^−1^; (AHOL1) for free LP to 33.9 µg mL^−1^ (C6) and 2.30 µg mL^−1^ (AHOL1) for LPN, and to 19.96 µg mL^−1^ (C6) and 1.70 µg mL^−1^ (AHOL1) for LP‐SF. At the highest concentration tested, LP‐SF induced complete C6 cell death; in AHOL1, 10 µg mL^−1^ sufficed for total loss of viability (Figure 3). Morphological changes at 72 h (Figures 4 and 5) accompanied the decrease in viability. The data are consistent with enhanced intracellular delivery mediated by nano/micro‐encapsulation and size‐dependent uptake, explaining the higher relative potency of LP‐SF and LPN versus LP. Given the high PDI of LP‐SF, we acknowledge heterogeneity; nonetheless, superior biological performance persisted within the tested range. Mechanistic assays (e.g., reactive oxygen species, Annexin V/PI, caspase‐3/7, ΔΨm, and γH2AX) will be prioritized to elucidate cell‐death pathways [47, 48, 51].

### 2.4 Safety Profile and Therapeutic Outlook

**FIGURE 6:**
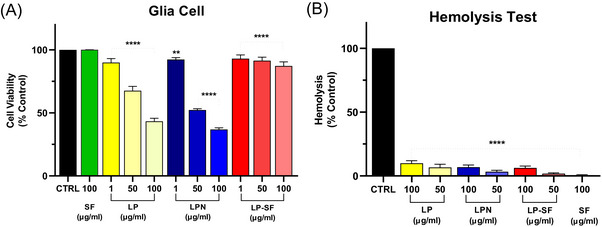
(A) Measurement of glial cell viability (3‐(4,5‐dimethylthiazol‐2‐yl)‐2,5‐diphenyltetrazolium bromide [MTT] assay) after 72 h in cells treated with lapachol (LP), lapachol nanoemulsion (LPN), silk fibroin‐based microemulsion (LP‐SF), and SF. Data are presented as mean ± SD (*n* = 3 independent experiments; three technical replicates each). ****p < 0.0001, compared to the untreated control group (CTRL) (analysis of variance [ANOVA], Tukey post‐test). (B) Hemolysis assay. Red blood cell suspensions were incubated with LP, LPN, and LP‐SF at concentrations of 10, 25, 50, 75, and 100 µg.mL^−1^ at 37°C. Triton‐X 100 (0.1%) was used as a positive control (100% hemoglobin release lysis). Data are presented as means (±SEM) of three independent experiments. ****p < 0.0001 versus Positive control (CTRL) (ANOVA, Tukey post‐test).

Neither formulation induced relevant hemolysis nor impaired primary‐glia viability (Figure 6 ), indicating a favourable in‐vitro therapeutic window [49]. Previous silk‐fibroin systems, such as curcumin‐loaded hydrogels and ICG‐bearing nanoparticles for photothermal therapy, demonstrate the dual role of fibroin in shielding the active ingredient and facilitating controlled, tumor‐selective delivery [50, 51]. Similarly, the gradual release of LP‐SF, combined with SF‐mediated interfacial organization, is likely to underlie the superior growth inhibition observed. Altogether, the physicochemical evidence, controlled‐release behavior, and enhanced cytotoxicity position LP‐SF as a promising candidate for glioma therapy. The next steps will focus on in vivo pharmacokinetics and availability, blood‐brain barrier permeability, and long‐term safety, with the aim of translating these in vitro findings. In parallel, we recognize inherent limitations of silk‐fibroin: (i) variability in source/processing (e.g., degumming, solvent, and β‐sheet content), which affects release and stability; (ii) potential immunogenicity due to residual sericin and endotoxin; and (iii) uncertainty regarding biodegradation in the central nervous system (CNS) over time. These limitations will be addressed.

**FIGURE 7:**
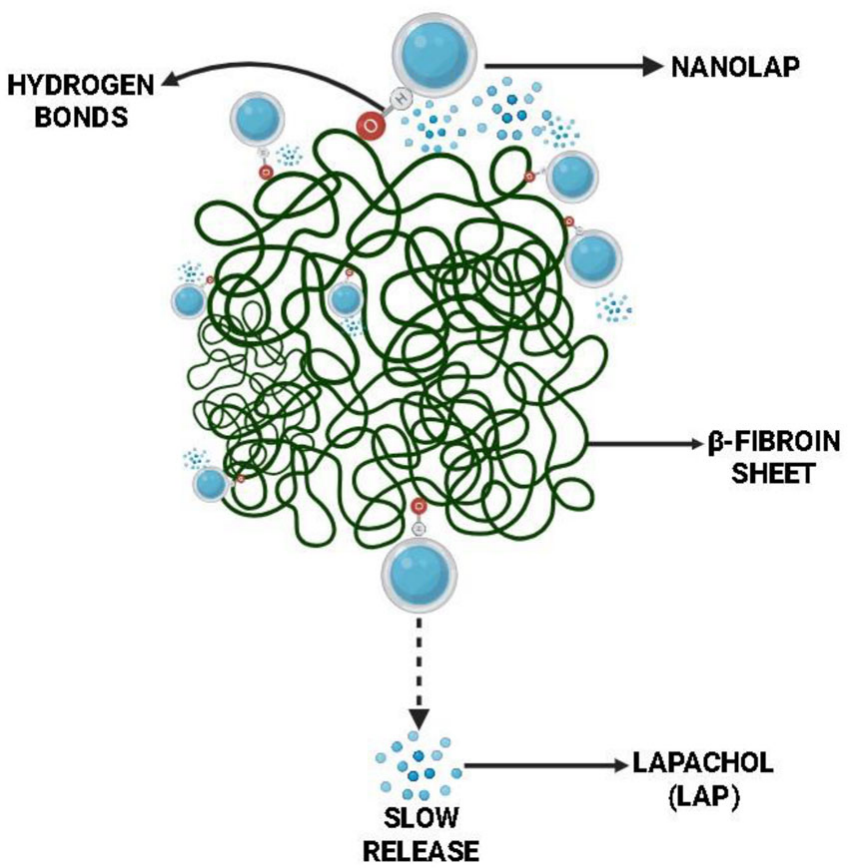
Schematic illustration of the diffusion‐controlled release mechanism of a silk fibroin‐based nanocarrier system.

## 3 Conclusions

The formulations evaluated significantly enhanced LP's antiproliferative activity in C6 and AHOL1 glioma cells. Both LP‐SF and LPN exhibited sustained drug release and significantly negative zeta potentials, indicating high colloidal stability and a more favorable *in vitro* therapeutic profile, compared to free LP. LP‐SF demonstrated superior biological performance and minimal hemolytic activity, without harmful effects in primary glial cells, supporting its potential as a promising delivery platform for LP in glioma treatment. Within the in vitro scope of this study, the next steps will focus on *vivo* validation, including pharmacokinetic and biodistribution studies, particularly with regard to blood‐brain barrier permeability and mechanistic assays aimed at consolidating the translational potential of this formulation, which may be promising for future studies using in vivo models. Furthermore, in the search for innovative and safe drug platforms against glioma, future strategies could include the co‐encapsulation of chemotherapeutic agents within the same LP‐SF structure, enabling combination therapies or synergistic effects.

## 4 Experimental

### 4.1 Plant Materials: Collection and Identification

LP was extracted from the powdered sawdust of *Tabebuia impetiginosa* wood using a 1% sodium carbonate solution. The wood was collected in Ferreira Gomes, Amapá, Brazil, on February 12, 2023, and the species was taxonomically identified by Dr. José Carlos Tavares Carvalho (Federal University of Amapá). Approximately 200 g of this sawdust was placed in a 2 L beaker containing 1 L of 1% sodium carbonate solution. The mixture was allowed to rest for 45 min. After this period, the solution was filtered, and 6 M hydrochloric acid (HCl) was slowly added to the filtrate, resulting in the formation of LP as a yellow precipitate. This precipitate was purified by column chromatography (CC) on silica gel, using a mixture of *n*‐hexane and ethyl acetate in a 7:3 ratio for elution. The purification process yielded 1.85 g of LP in the form of yellow crystals. The isolated product was characterized using GC–MS and ^1^H and ^13^C NMR, along with Fourier‐transform infrared spectroscopy. The data obtained were compared with those of the existing literature to confirm the identity of the product [43].

### 4.2 Gas Chromatography‐MS

Compound analysis was performed on a Shimadzu GCMS‐QP2010 with an AOC‐20i autosampler, operating in EI mode at 70 eV with a scan range of *m/z* 50–550. Separation was achieved on a fused‐silica capillary RTX‐5MS column (30 m × 0.25 mm × 0.25 µm) using helium as the carrier gas (column flow 1.03 mL min^−1^). Samples were dissolved in dichloromethane at 2 µg mL^−1^, and 1.0 µL was injected in split 1:10 mode. Temperatures were: injector 210°C and detector 250°C. The oven program was 130°C for 3 min, ramp 5°C min^−1^ to 290°C, then hold 5 min at 290°C. The total run time was 40 min.

### 4.3 Nuclear Magnetic Resonance

^1^H NMR spectra were recorded on an Agilent Technologies Premium 400/54 (400 MHz) with a shielded probe at 25°C. Samples were dissolved in CDCl_3_, and chemical shifts (δ) are reported in ppm referenced to TMS as the internal standard. When TMS was not added, residual solvent signals were used as reference (δ 7.26 ppm for CDCl_3_ and for ^13^C, δ 77.16 ppm). Coupling constants (*J*) are given in Hz, and multiplicities follow standard notation: singlet (s), doublet (d), triplet (t), quartet (q), multiplet (m), and broad singlet (br s).

### 4.4 Preparation of SF Solution

The SF solution was prepared following established protocols [47]. Briefly, 3.0 g of silkworm cocoon material were cut into small pieces and immersed in 500 mL of 2% (w/v) Na_2_CO_3_. The mixture was heated to 100°C under magnetic stirring for 30 min, yielding a fibrous material. The fibers were then washed with distilled water three times (1000 mL each) and dried in an oven at 70°C for 24 h. To dissolve SF, 50 mL of an H_2_O:EtOH:CaCl_2_ solution at a molar ratio of 8:2:1 (equivalent to CaCl_2_:EtOH:H_2_O = 1:2:8) was added. The mixture was maintained at 80°C for 6 h with continuous magnetic stirring. The resulting solution was dialyzed at room temperature for 3 days, with water replacement every 24 h. To remove coarse particles, the SF solution was centrifuged at 6000 rpm for 10 min and then stored at 10°C.

### 4.5 Preparation of LPNs With Polysorbate 85

LP stock solutions (LSS) were prepared using 96% (v/v) ethanol to create the oil phases (OP) necessary for formulating nanodispersions, with each component (either a bioactive compound or a non‐ionic surfactant) at a concentration of 4 mg/mL. The components included LP, a combination of LP and polysorbate 85 (LP85). Each organic phase was added at a rate of 500 µL, with the OP being slowly dripped into the aqueous phase while maintaining continuous magnetic stirring.

### 4.6 DLS Analysis

The nanodispersion was characterized by DLS using a Zetasizer NanoZS (Malvern, UK). The scattering angle was set at 173°, with deionized water as the dispersing medium. Measurements were performed in triplicate at 25°C (room temperature).

### 4.7 Preparation of LP‐SF

P‐SF was prepared from an LP stock solution at 200 mg.mL^−1^ in isobutanol (*i*‐BuOH). The system comprised 6% of an SF solution (5%, w/w), 2% Tween 80 (T80), 0.13% of the LP stock solution, and water to a final mass of 3 g. The mixture was then sonicated using an LGU‐LUC‐180 ultrasonic processor at 28/40 kHz for 2 min at 2% power, with 10 s pulses.

### 4.8 Droplet Size Analysis of the Emulsion

Droplet size, zeta potential, and PDI were determined by photon correlation spectroscopy using a Zetasizer 5000 (Malvern Instruments, UK). Each emulsion was diluted using ultrapure Milli‐Q water. Measurements were performed in triplicate (Table 2).

**TABLE 2:** Dynamic light scattering analysis of lapachol nanoemulsion (LPN) and silk fibroin‐based microemulsion (LP‐SF).

Emulsion	Zeta size (nm)	Polydispersity index	Potential zeta (mV)
LPN	88.3 ± 1.17	0.29 ± 0.02	−0.834 ± 0.025
LP‐SF	469.86 ± 3.98	0.92 ± 0.09	−0933 ± 0.298

### 4.9 In Vitro Drug Release Analysis

In vitro release of the LP‐SF was performed using the dialysis bag method. Experiments were conducted at 37°C with PBS as the release medium. A dialysis bag (MWCO 12 000–14 000 Da, Sigma‐Aldrich, St. Louis, MO, USA) containing 2 mL of sample was placed in contact with 100 mL of release medium under moderate magnetic stirring, ensuring sink conditions. At predetermined time points (0, 1, 2, 3, 4, 5, 6, 7, 8, and 24 h), 1 mL of the medium was withdrawn and replaced with an equal volume of fresh medium. LP‐SF concentration was quantified by UV‐Vis at 274 nm. For comparison, diffusion of unencapsulated LP from a 50:50 (v/v) hydroalcoholic mixture at the same concentration was also evaluated. Three independent experiments were performed for each formulation. Data were analyzed by mathematical modeling using KinetDS (version 3.0, Kraków, Poland) to better understand LP release from LP‐SF. The model that best described the profile was selected based on the correlation coefficient (r).

### 4.10 Cell Culture and Experimental Conditions

The C6 rat glioma cell line (RRID: CVCL_0194) was obtained from the Rio de Janeiro Cell Bank (BCRJ, Brazil). The human glioma cell line, AHOL1 (RRID: CVCL_XH23), was kindly provided by Dr. Edivaldo Herculano de Oliveira (Evandro Chagas Institute, Brazil). Both cell lines were maintained at 37°C in a humidified incubator with 5% CO_2_/95% air, in Dulbecco's Modified Eagle Medium (DMEM) supplemented with 10% fetal bovine serum (FBS), 100 U mL^−1^ penicillin, and 100 µg mL^−1^ streptomycin. Primary glial cultures were prepared from retinas of 7‐day‐old chick embryos, which were dissected, incubated with trypsin/EDTA, and mechanically dissociated in DMEM + 10% FBS. Cells were centrifuged at 1500 rpm for 5 min and cultured for 14 days. All experiments were performed on cells in the logarithmic growth phase.

### 4.11 Cell Cytotoxicity Assay

The cytotoxicities of LP, LPN, and LP‐SF were assessed using the MTT assay, a water‐soluble tetrazolium salt that viable cells reduce to insoluble purple formazan crystals. Cells were seeded into 24‐well plates (1 × 10⁵ cells/well). Upon reaching 90% confluence, cells were treated with LP, LPN, and LP‐SF at 10, 25, 50, 75, and 100 µg mL^−1^ for 24, 48, and 72 h. Concentrations were chosen based on prior studies showing dose‐dependent effects of related compounds in tumor cell lines. After incubation, cells were washed with PBS, and MTT solution at 0.5 mg mL^−1^ (50 µL/well) was added and incubated for 3 h. Subsequently, 100 µL of DMSO was added to dissolve the formazan. Absorbance was read at 570 nm using a microplate reader (BIO‐RAD Model 450).

### 4.12 In Vitro Hemolysis Assay

The in vitro safety of LP, LPN, and LP‐SF was assessed by a hemolysis test. Rat blood was collected and centrifuged at 3000 rpm for 5 min. Red blood cells (RBCs) were washed and resuspended to a 2% (v/v) suspension in 0.85% (w/v) NaCl saline. LP, LPN, and LP‐SF at 10, 25, 50, 75, and 100 µg mL^−1^ were incubated with the RBC suspension (1:1, v/v) for 3 h at room temperature under constant agitation. Triton X‐100 (0.1%) served as the positive control (100% hemolysis/hemoglobin release), and 0.9% saline as the negative control. After incubation, samples were centrifuged (3,000 rpm, 5 min), and hemoglobin release was measured by UV‐Vis spectrophotometry at 450 nm. Healthy glial cells were used as controls.

### 4.13 Statistical Analysis

Data were pre‐processed by inspecting outliers and testing normality with the Shapiro–Wilk test. Results are reported as means ± SD. Sample sizes were: release, *n* = 3 independent batches; cytotoxicity, *n* = 3 independent experiments with 3 technical replicates per condition; hemolysis, *n* = 3 animals. Group comparisons were performed using one‐way ANOVA (two‐sided, α = 0.05), followed by Tukey's post hoc test when parametric assumptions were met; otherwise, Kruskal–Wallis with Dunn's adjustment was applied. Analyses were performed in GraphPad Prism 8.0.

## Author Contributions

**Investigation**: Jardel P. Queiroz, Eline Gomes Santos, and Fabrício Holanda; **Data curation**: Jardel P. Queiroz, Eline Gomes Santos, and Victor Marinho; **Resources**: Irlon M. Ferreira, Fábio R. Oliveira, and Edilene Oliveira da Silva; **Visualization**: Jardel P. Queiroz., Eline Gomes Santos, Fabrício Holanda, Fábio R. Oliveira, Barbarella M. Macchi, and Caio P. Fernandes; **Writing – original draft preparation**: Jardel P. Queiroz, Irlon M. Ferreira, and José Luiz M. Nascimento; **Writing – review and editing**: Barbarella M. Macchi, Edilene Oliveira da Silva, Irlon M. Ferreira, and José Luiz M. Nascimento; **Supervision**: José Luiz Martins do Nascimento, José Carlos T. Carvalho, Caio P. Fernandes, and Barbarella M. Macchi; **Conceptualization, project administration and funding acquisition**: José Luiz M. Nascimento. All authors have read and agreed to the published version of the manuscript. All authors contributed to the article.

## Funding

This research was funded by Conselho Nacional de Desenvolvimento Científico e tecnológico (CNPQ Grant Number 444009/2024‐8) and Fundação de Amparo à Pesquisa do Estado do Pará (FAPESPA, Grant Number 063/2020).

## Conflicts of Interest

The authors declare no conflicts of interest.

## Ethics Statement

All animal procedures were approved by the Animal Ethics Committee of the Federal University of Pará (CEUA/UFPA, protocol 80/15) and carried out in accordance with applicable national and institutional guidelines, including Brazilian Law 11.794/08, CONCEA regulations, and the NIH Guide for the Care and Use of Laboratory Animals. No human samples were used.

## Data Availability

All data generated or analized during this study are included in this published article
